# Supplementary material for: High-throughput single-cell chromatin accessibility CRISPR screens enable unbiased identification of regulatory networks in cancer
Source: Nat Commun. 2021 May 20;12:2969. doi: 10.1038/s41467-021-23213-w (PMC8137922; doi:10.1038/s41467-021-23213-w)
Supplement: Supplementary file 1 — Supplementary Information [file 41467_2021_23213_MOESM1_ESM.pdf]

Supplementary Figure 1

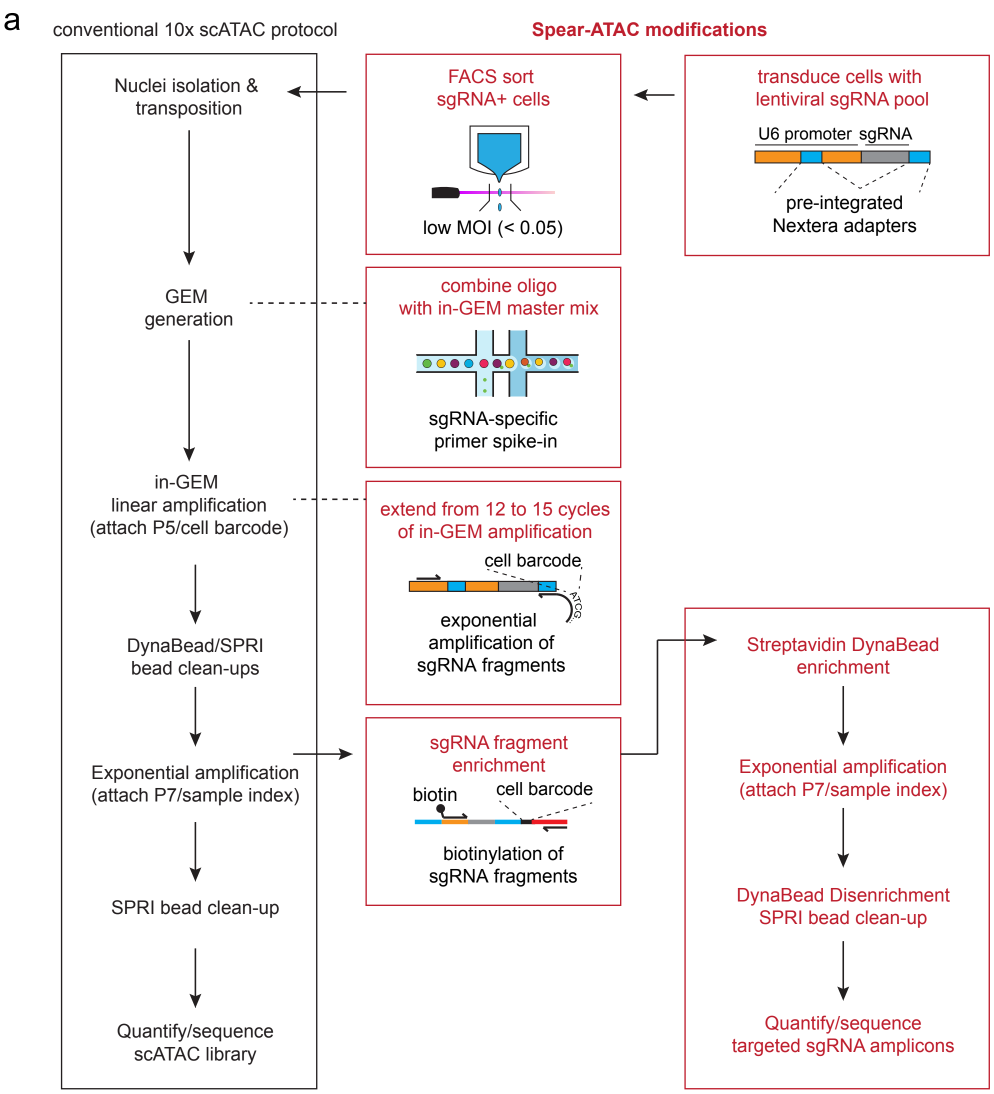

Supplementary Figure 1. Outline of Spear-ATAC protocol.

a. Cells are transduced with a lentiviral sgRNA flanked by Read1/Read2 Nextera adapters, FACS sorted to exclude sgRNA-negative cells, and processed for scATAC-seq using a modified 10x Genomics droplet-based protocol. In brief, nuclei are isolated, transposed, and gel bead emulsions (GEMs) are made with individual nuclei re-suspended in Nuclei Resuspension Buffer combined with an enzymatic master mix containing a sgRNA-specific primer. GEMs are immediately subjected to in-GEM linear amplification of scATAC-seq fragments, while sgRNA fragments are subjected to exponential amplification using the sgRNA-specific primer as a Forward primer. The number of cycles of in-GEM amplification has been extended from 12 cycles (original 10x protocol) to 15 cycles (Spear-ATAC protocol). Following a series of clean-ups and exponential amplification to attach flow-cell adapters (P7) and sample indices to the ATAC-seq fragments, sgRNA fragments are specifically enriched using a biotin-conjugated oligo specific to the sgRNA fragments, and then amplified and sequenced separately.

Supplementary Figure 2

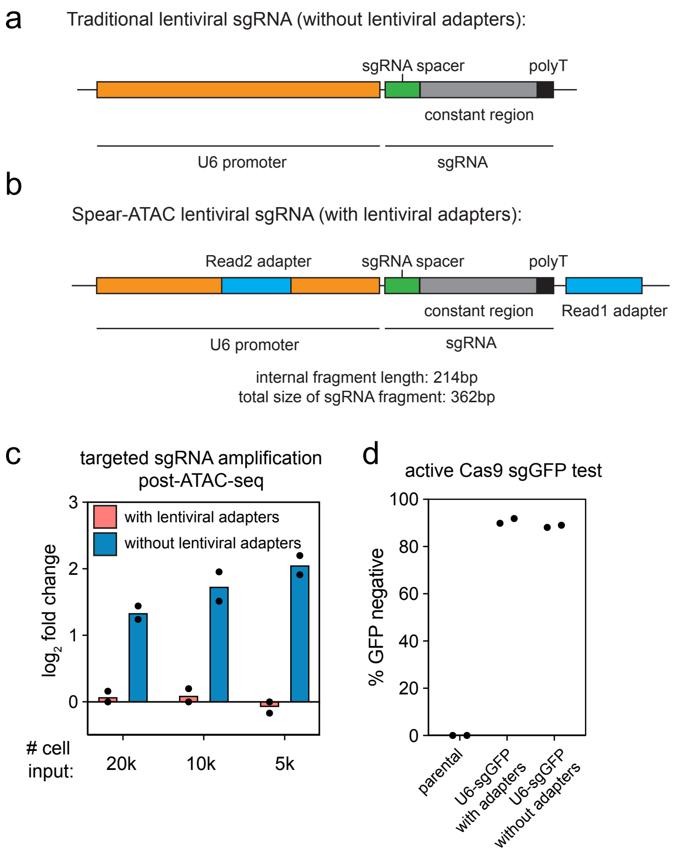

Supplementary Figure 2. Flanking a lentiviral sgRNA spacer with Read1/Read2 adapters increases sgRNA fragment capture efficiency following ATAC-seq.

a. Schematic of a traditional lentiviral sgRNA.

b. Schematic of a modified Spear-ATAC lentiviral sgRNA. sgRNA spacer sequence is flanked by Read1/Read2 Nextera adapters, obviating the need for tn5 transposase to randomly insert sequencing adapters nearby the sgRNA sequence during an ATAC-seq transposition reaction. The Read2 adapter is inserted in a flexible region of the U6 promoter to decrease the total length of the sgRNA fragment within the scATAC-seq library.

c. Log_2 fold change in sgRNA fragments amplified following bulk ATAC-seq reactions of cells that were previously transduced with lentiviral sgRNAs with and without Nextera adapters flanking the sgRNA region. Reactions were performed with the indicated numbers of input cells. n=2 biologically independent samples examined over 2 independent experiments. Source data is available in Supplementary Data 9.

d. % GFP negative cells following lentiviral transduction of self-targeting sgGFP-GFP constructs with and without Nextera adapters flanking the sgRNA region. n=2 biologically independent samples examined over 2 independent experiments. Source data is available in Supplementary Data 9.

Supplementary Figure 3

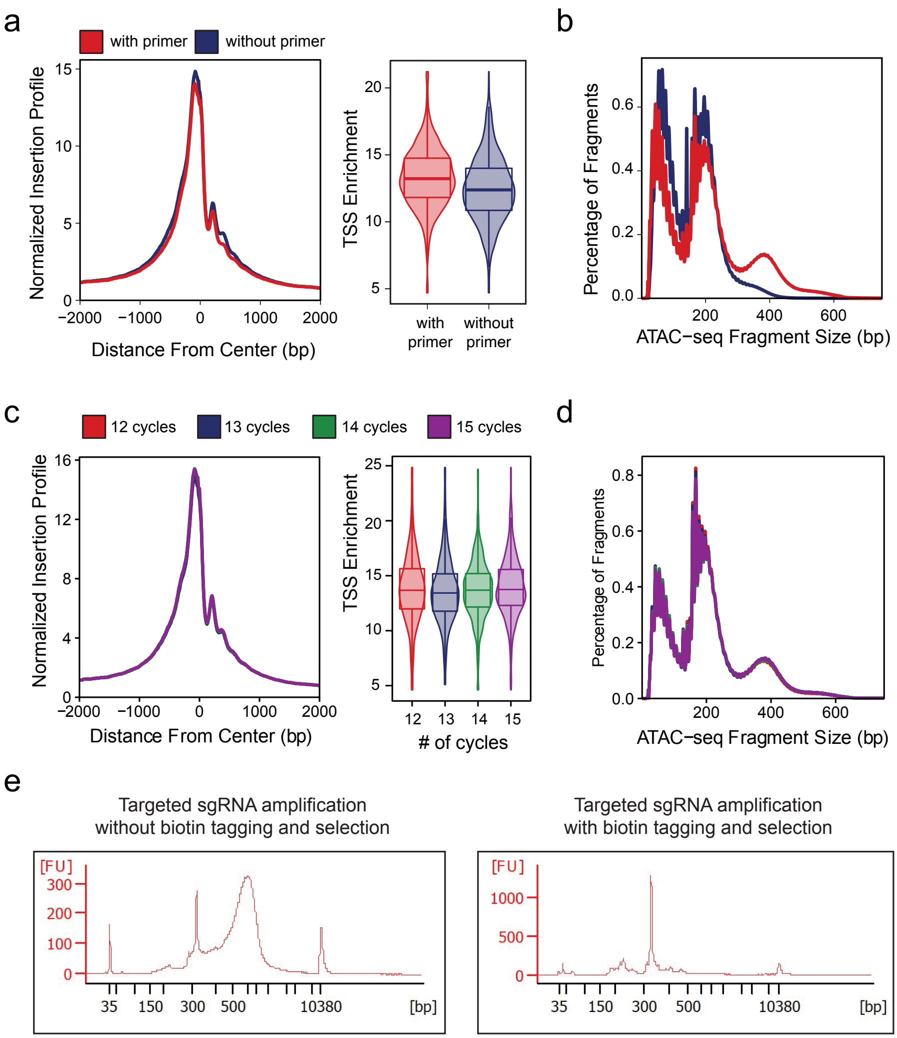

Supplementary Figure 3. Spear-ATAC modifications to the 10x scATAC-seq protocol do not hurt scATAC quality.

a-b. scATAC-seq quality control metrics with and without a sgRNA-specific primer spiked into the enzymatic master mix during GEM formation on the 10x Controller. Insertion profiles (left a), TSS enrichment (right a), and average fragment distribution (b) in each sample is shown. Box-whisker plot; lower whisker is the lowest value greater than the 25% quantile minus 1.5 times the interquartile range (IQR), the lower hinge is the 25% quantile, the middle is the median, the upper hinge is the 75% quantile and the upper whisker is the largest value less than the 75% quantile plus 1.5 times the IQR. N=4902 cells examined without a primer spiked in and N=5888 cells examined with a primer spiked in over 1 independent experiment.

c-d. scATAC-seq quality control metrics after 12, 13, 14, and 15 cycles of in-GEM linear amplification. Insertion profiles (left c), TSS enrichment (right c), and average fragment distribution (d) in each sample is shown. Box-whisker plot; lower whisker is the lowest value greater than the 25% quantile minus 1.5 times the interquartile range (IQR), the lower hinge is the 25% quantile, the middle is the median, the upper hinge is the 75% quantile and the upper whisker is the largest value less than the 75% quantile plus 1.5 times the IQR. N=1114 cells examined with 12 cycles of PCR amplification, N=936 cells examined with 13 cycles of PCR amplification, N=888 cells examined with 14 cycles of PCR amplification, and N=961 cells examined with 15 cycles of PCR amplification over 1 independent experiment.

e. Biotin tagging and selection increases the specificity of targeted sgRNA amplification, as seen by the fragment distribution of products without (left) and with (right) the first biotin tagging step.

Supplementary Figure 4

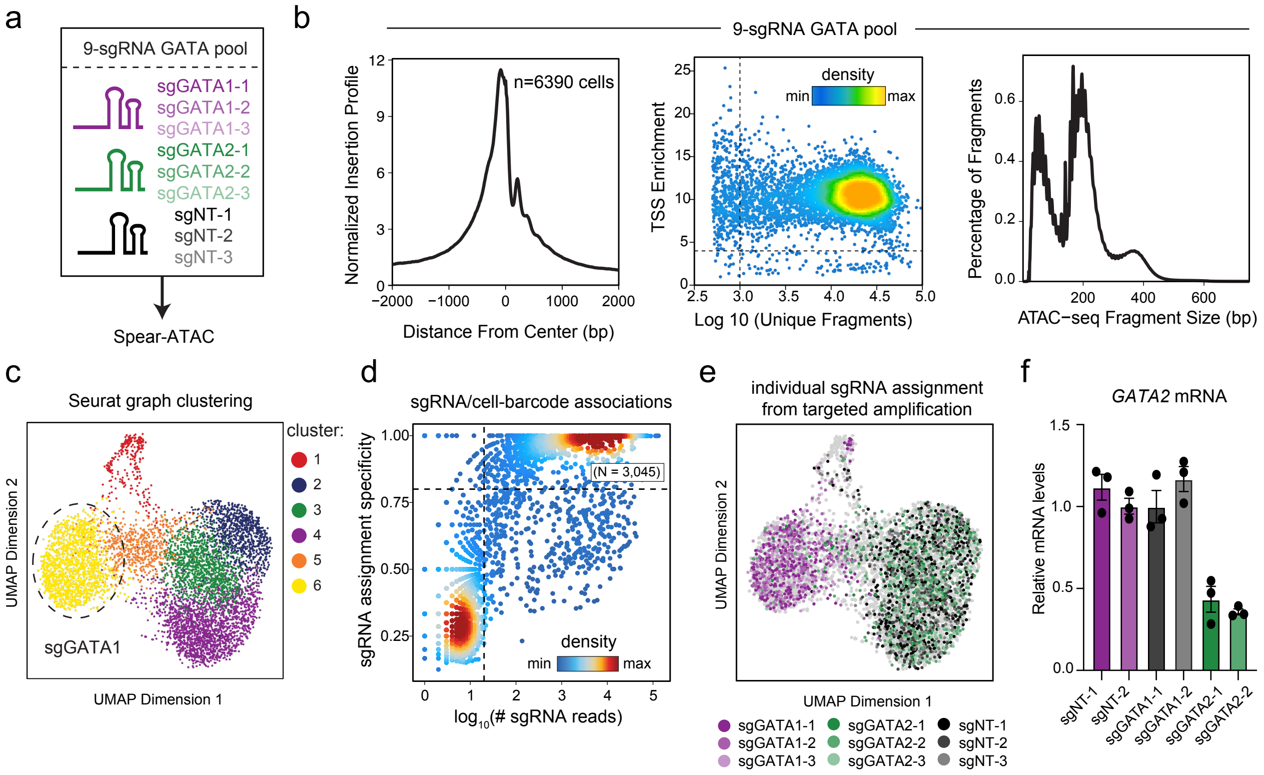

Supplementary Figure 4. Quality control metrics and sgRNA assignment methods for 9-sgRNA GATA pool.

a. Schematic of sgRNAs in the first proof-of-principle 9-sgRNA pool.

b. scATAC-seq quality control metrics for the proof-of-principle 9-sgRNA GATA pool. Average TSS enrichment (left), individual TSS enrichment x $\log_{10}$ number of unique fragments per cell (middle), and average insertion profiles (right inset) for the 6,390 nuclei captured.

c. UMAP of Spear-ATAC chromatin accessibility profiles (N = 6,390) for the 9-sgRNA K562 screen colored by Seurat graph clustering in ArchR.

d. Specificity to a single sgRNA spacer sequence and number of sgRNA reads ($\log_{10}$) following targeted amplification for each cell analyzed.

e. UMAP of Spear-ATAC chromatin accessibility profiles for the 9-sgRNA K562 screen colored by the individual sgRNA.

f. Relative GATA2 mRNA expression in K562;dCas9-KRAB cells expressing the indicated sgRNAs (sgNT-1, sgNT-2, sgGATA1-1, sgGATA1-2, sgGATA2-1, sgGATA2-2). n= 3 biologically independent samples examined over 2 independent experiments. Error bars represent standard error of the mean. Source data is available in Supplementary Data 9.

Supplementary Figure 5

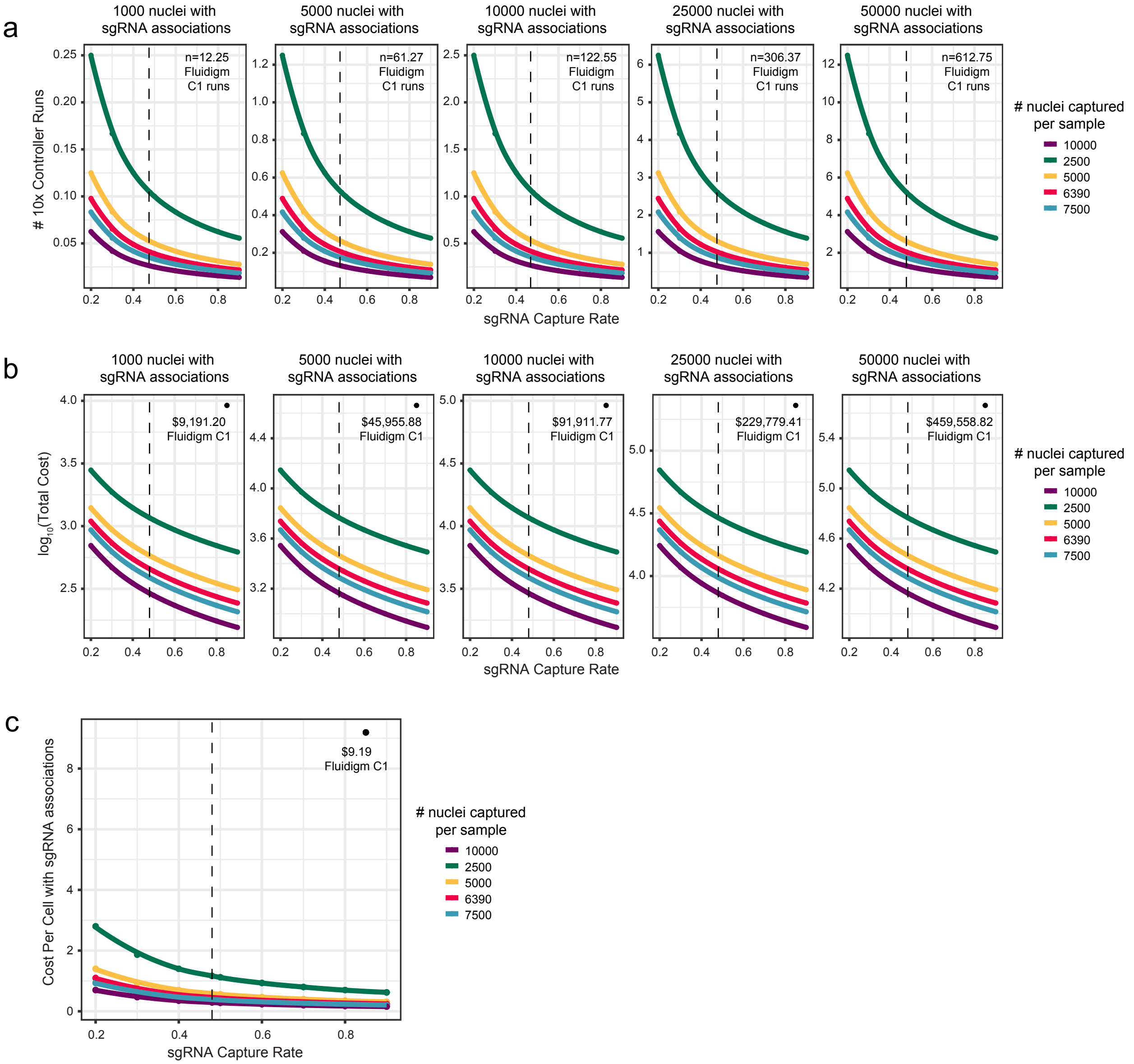

Supplementary Figure 5. Cost and throughput comparisons between Spear-ATAC and Perturb-ATAC.

a. Number of 7-minute 10x Controller runs required (y-axis) to process the indicated number of nuclei with sgRNA associations (top) given a specific sgRNA capture rate (x-axis) and number of nuclei captured per sample. Up to 8 samples can be processed on a single 10x Controller run, which is the limiting factor for throughput of Spear-ATAC. The corresponding number of Fluidigm C1 runs using Perturb-ATAC required (given an 85% sgRNA capture rate and 96 nuclei captured per run) is indicated in the top right. In the pilot experiment (Figure 1), 6,390 nuclei were captured (shown in pink) with a 48% sgRNA capture rate (indicated using the dashed line).

b. $\log_{10}$(Total Cost) (y-axis) in U.S. dollars ($) of the experiment to process the indicated number of nuclei with sgRNA associations (top) given a specific sgRNA capture rate (x-axis) and number of nuclei captured per sample. The cost required to acquire similar data using Perturb-ATAC (given an 85% sgRNA capture rate and 96 nuclei captured per run) is indicated in the top right. In the pilot experiment (Figure 1), 6,390 nuclei were captured (shown in pink) with a 48% sgRNA capture rate (indicated using the dashed line).

c. Cost per cell with sgRNA associations (y-axis) in U.S. dollars ($) given a specific sgRNA capture rate (x-axis) and number of nuclei captured per sample. The cost required to acquire similar data using Perturb-ATAC (given an 85% sgRNA capture rate and 96 nuclei captured per run) is indicated in the top right.

Supplementary Figure 6

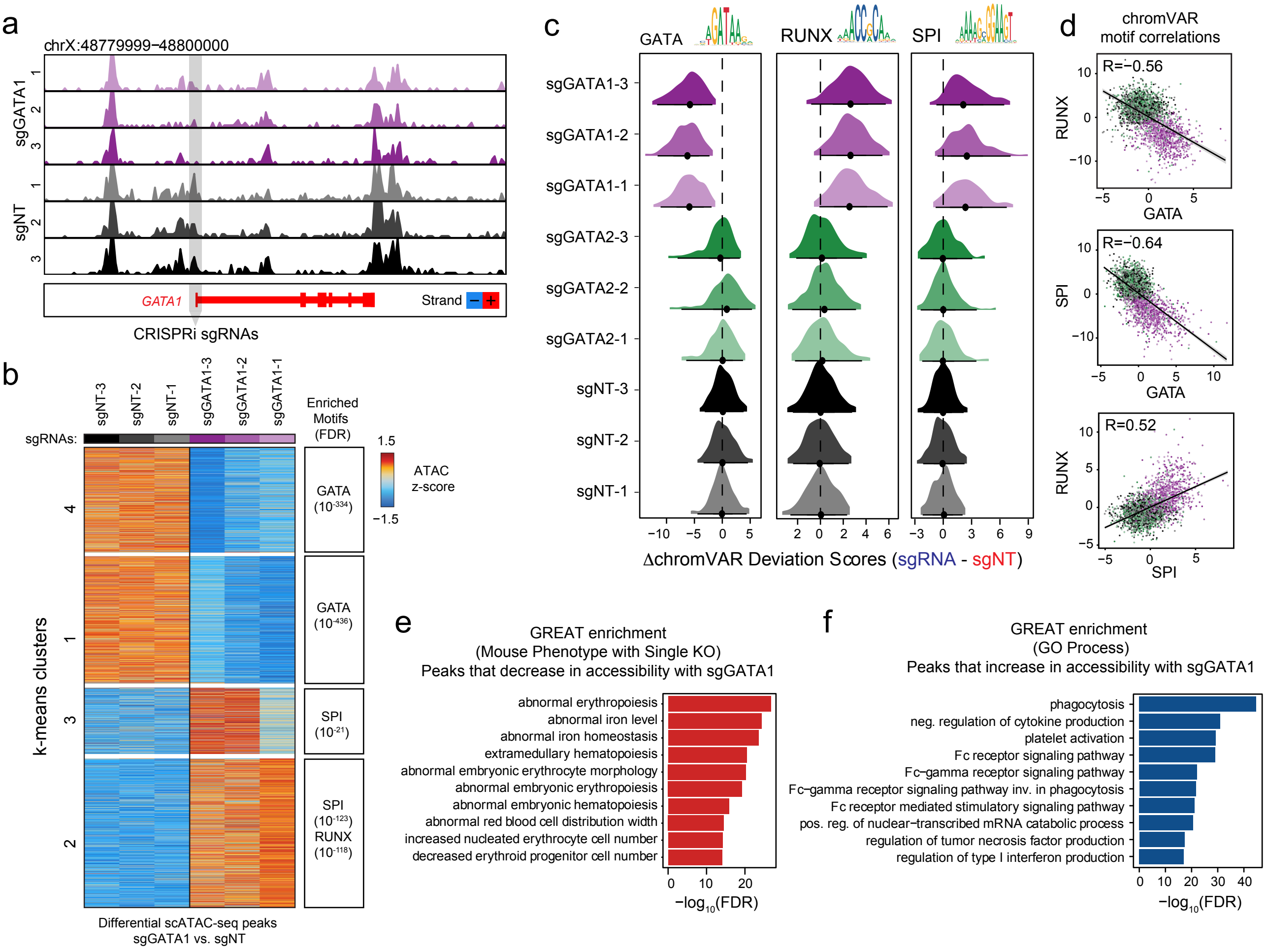

Supplementary Figure 6. GATA1 maintains accessibility at genomic regions important for erythropoiesis.

a. Pseudo-bulk ATAC-seq track at the GATA1 locus for individual sgRNAs of sgGATA1 and sgNT. Light grey box indicates the region targeted by sgGATA1-1, sgGATA1-2, and sgGATA1-2 CRISPRi sgRNAs.

b. Heatmap of chromatin peak accessibility for each individual sgRNA for both sgGATA1 and sgNT using the top differential scATAC-seq peaks identified from ArchR. Each row represents a z-score of $\log_2$ normalized accessibility within each group using scATAC-seq. (Right) Transcription factor hypergeometric motif enrichment with FDR indicated in parentheses.

c. Ridge plot for chromVAR deviations (normalized to the median sgNT) of individual cells for (Left) GATA, (Middle) RUNX and (Right) SPI. For each ridge, black dot represents median and bar extends from the 2.5th to 97.5th quantiles.

d. ChromVAR motif correlations for the indicated transcription factor families (RUNX, SPI, or GATA). Pearson correlation (R) is indicated in the top left corner. Each individual point represents a single cell. Cells are colored based on their sgRNA genotype.

e. GREAT enrichment of peaks that decrease in accessibility with sgGATA1. Enrichments are taken from the Mouse Phenotype with Single KO (Knock-out) dataset.

f. GREAT enrichment of peaks that increase in accessibility with sgGATA1. Enrichments are taken from the GO Process dataset.

Supplementary Figure 7

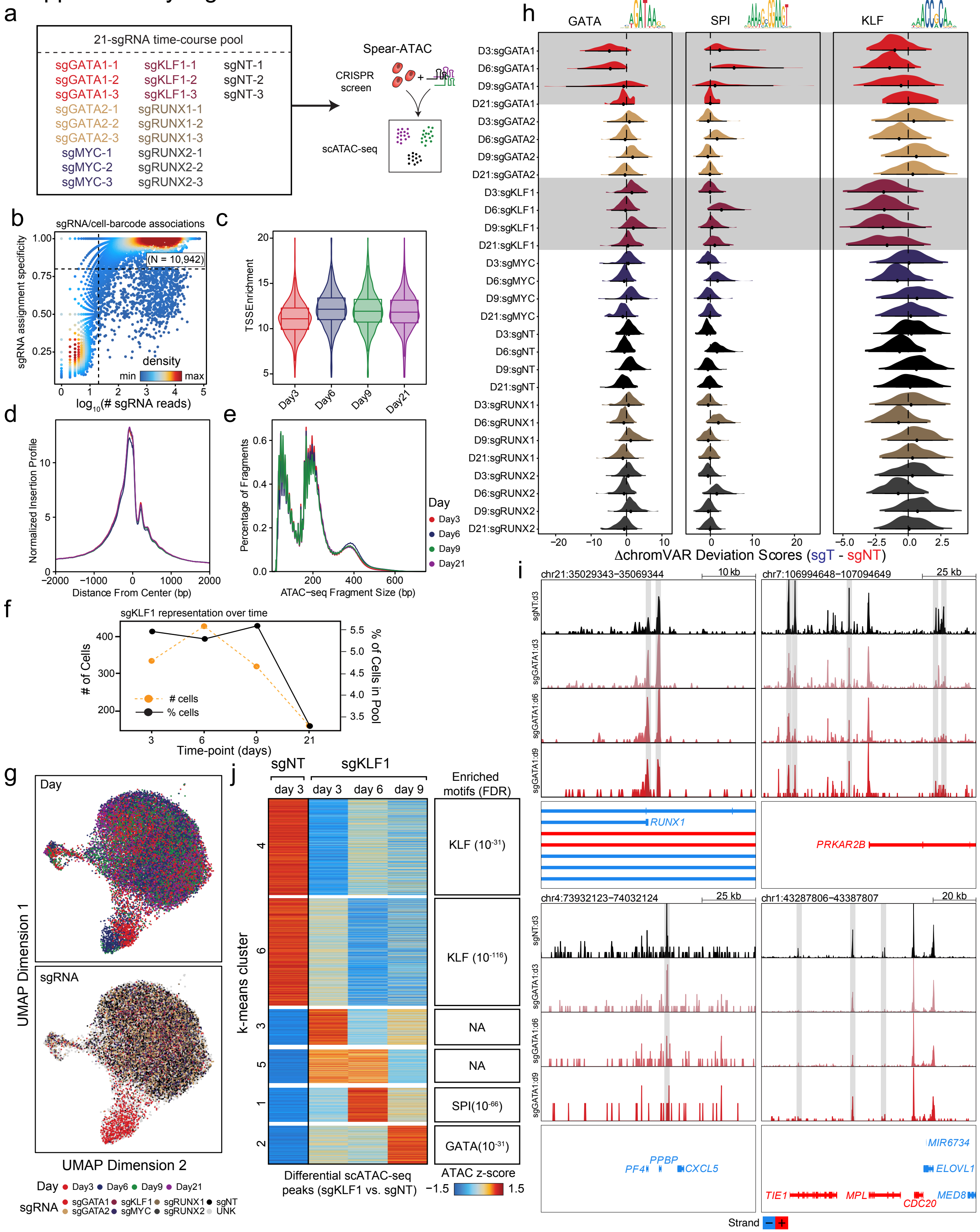

Supplementary Figure 7. Spear-ATAC increases the throughput of time-course screens to understand the timing of effects following transcription factor perturbations.

a. Schematic of 21 sgRNAs used in the Spear-ATAC K562 time-course screen.

b. Specificity to a single sgRNA spacer sequence and number of sgRNA reads (log_{10}) following targeted amplification for each cell analyzed.

c. TSS enrichment scores per single cell across all Spear-ATAC K562 time-course time points. Box-whisker plot; lower whisker is the lowest value greater than the 25% quantile minus 1.5 times the interquartile range (IQR), the lower hinge is the 25% quantile, the middle is the median, the upper hinge is the 75% quantile and the upper whisker is the largest value less than the 75% quantile plus 1.5 times the IQR. N=24,856 cells examined over 4 independent experiments.

d. Aggregate TSS enrichment scores across all Spear-ATAC K562 time-course time points.

e. Aggregate ATAC-seq fragment size distributions across all Spear-ATAC K562 time-course time points.

f. Aggregate ATAC-seq fragment size distributions across all Spear-ATAC K562 time-course time points.

g. Change in sgKLF1 representation over time, represented by the number of cells analyzed per time-point (orange) and the % of cells in the total pool (black).

h. Ridge plot for chromVAR deviations (normalized to the median sgNT) of individual cells across each time point and target sgRNA for (Left) GATA, (Middle) SPI and (Right)

KLF. For each ridge, black dot represents median and bar extends from the 2.5th to 97.5th quantiles.

i. Pseudo-bulk ATAC-seq track at the (Top Left) RUNX1, (Top Right) PRKAR2B, (Bottom Left) PPBP and (Bottom Right) MPL locus for sgGATA1 (day3, day6, day9, and day21) and sgNT cells (day3). Light grey box indicates peak regions that changed in accessibility in the sgGATA1 vs sgNT cells.

Supplementary Figure 8

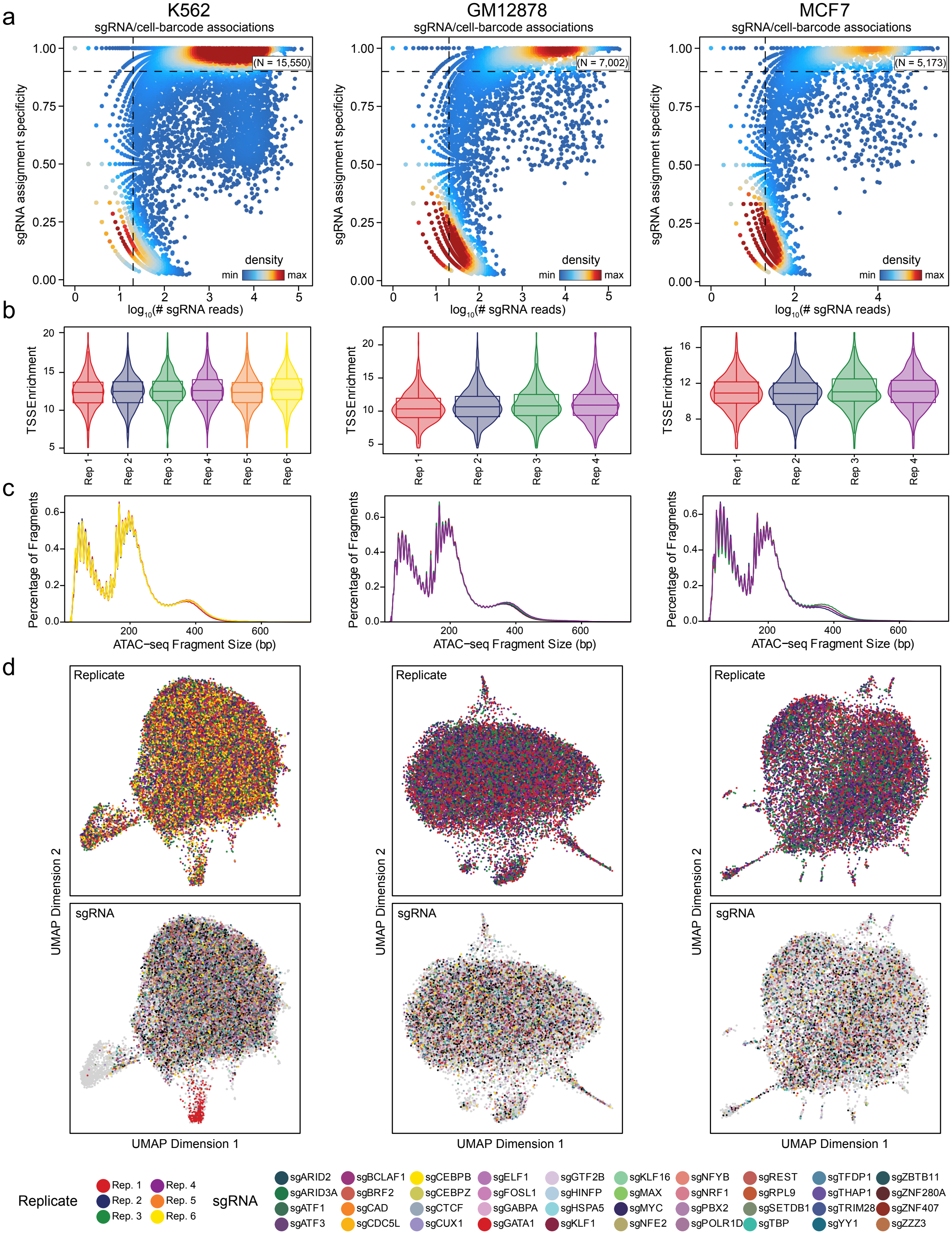

Supplementary Figure 8. Quality control metrics for Spear-ATAC transcription factor screens in K562;dCas9-KRAB, GM12878;dCas9-KRAB, and MCF7;dCas9-KRAB cell lines.

a. Specificity to a single sgRNA spacer sequence and number of sgRNA reads (log10) following targeted amplification for each cell analyzed for the (Left) K562, (Middle) GM12878 and (Right) MCF7 large screens.

b. TSS enrichment scores per single cell across the (Left) K562, (Middle) GM12878 and (Right) MCF7 large screen replicates. Box-whisker plot; lower whisker is the lowest value greater than the 25% quantile minus 1.5 times the interquartile range (IQR), the lower hinge is the 25% quantile, the middle is the median, the upper hinge is the 75% quantile and the upper whisker is the largest value less than the 75% quantile plus 1.5 times the IQR. N=32,832 K562;dCas9-KRAB cells, N=22,489 GM12878;dCas9-KRAB cells, and N=18,025 MCF7;dCas9-KRAB cells examined over 14 independent replicates.

c. Aggregate ATAC-seq fragment size distributions across the (Left) K562, (Middle) GM12878 and (Right) MCF7 large screen replicates.

d. UMAP of Spear-ATAC chromatin accessibility profiles for the (Left) K562, (Middle) GM12878 and (Right) MCF7 large screens. This UMAP is colored by (Top) experimental replicate and (Bottom) assigned sgRNA.

Supplementary Figure 9

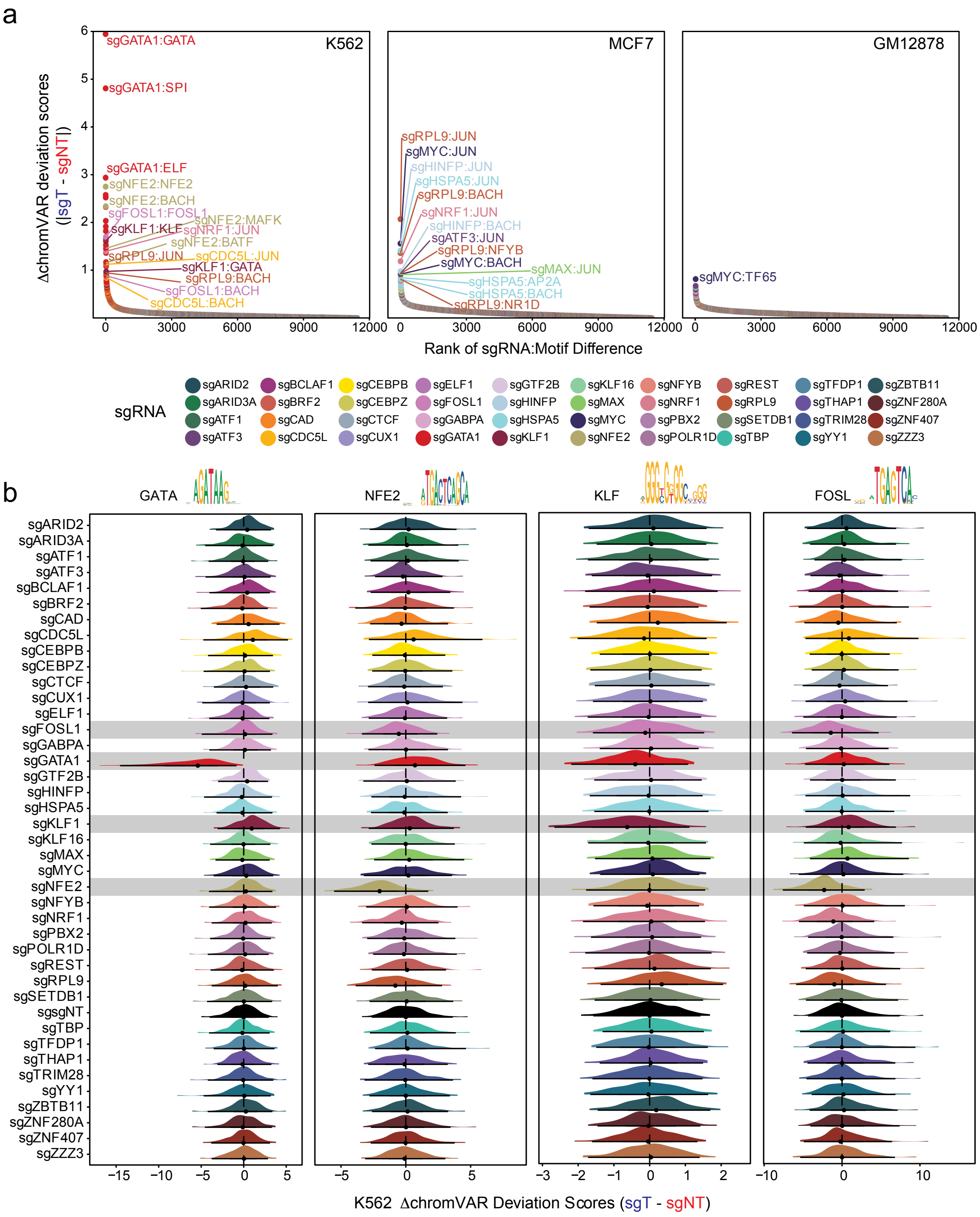

Supplementary Figure 9. Transcription factor motif accessibility changes following perturbations in K562;dCas9-KRAB, GM12878;dCas9-KRAB, and MCF7;dCas9-KRAB cell lines.

a. Rank ordered plot of sgRNA:TF perturbations to identify top hits for the (Left) K562, (Middle) GM12878 and (Right) MCF7 large screens.

b. Ridge plot for chromVAR deviations (normalized to the median sgNT) of individual cells for (Left) GATA, (Middle Left) NFE2, (Middle Right) KLF and (Right) FOSL across the K562 large screen. For each ridge, black dot represents median and bar extends from the 2.5th to 97.5th quantiles.

Supplementary Figure 10

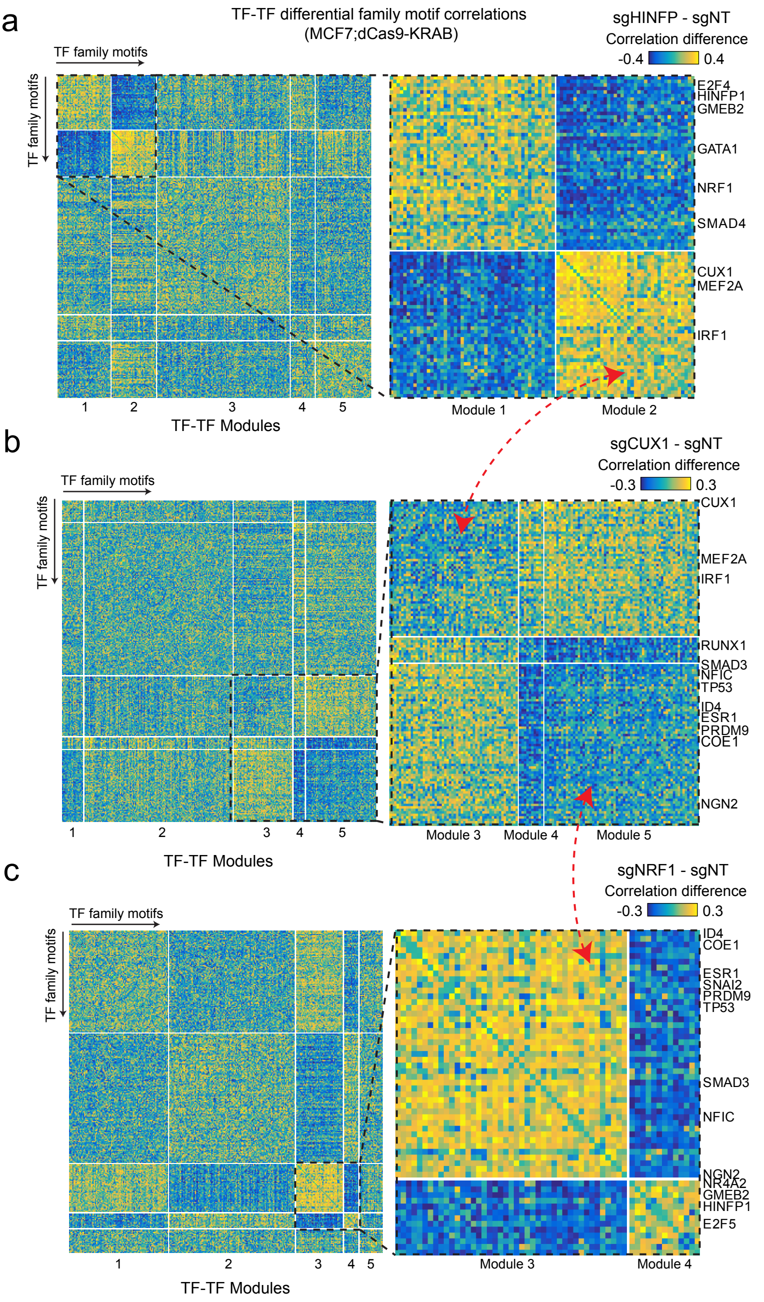

Supplementary Figure 10. Patterns of differential transcription factor motif correlations in MCF7;dCas9-KRAB cells following knockdown of HINFP, CUX1, and NRF1.

a. (Left) Heatmap of the differences between sgHINFP targeting and non-targeting cells for all TF-TF motif accessibility correlations grouped into 5 different modules in the MCF7 large screen. (Right) Zoomed in heatmap of modules 1 and 2 highlighting transcription factors with largely perturbed TF-TF motif accessibility relationships.

b. (Left) Heatmap of the differences between sgCUX1 targeting and non-targeting cells for all TF-TF motif accessibility correlations grouped into 5 different modules in the MCF7 large screen. (Right) Zoomed in heatmap of modules 1 and 2 highlighting transcription factors with largely perturbed TF-TF motif accessibility relationships.

c. (Left) Heatmap of the differences between sgNRF1 targeting and non-targeting cells for all TF-TF motif accessibility correlations grouped into 5 different modules in the MCF7 large screen. (Right) Zoomed in heatmap of modules 1 and 2 highlighting transcription factors with largely perturbed TF-TF motif accessibility relationships.

Supplementary Notes

Supplementary Note 1. Protocol for cloning Spear-ATAC plasmids.

This supplementary note contains a full protocol for cloning Spear-ATAC plasmids.

Supplementary Note 2. Protocol for making Spear-ATAC lentivirus and sample protocol for transducing cell lines of interest.

This supplementary note contains a full protocol for making lentivirus from Spear-ATAC plasmids. In addition, we include a sample protocol for transducing cell lines of interest, although this step will have to be optimized if a user is using cell lines other than K562;dCas9-KRAB, GM12878;dCas9-KRAB, or MCF7;dCas9-KRAB.

Supplementary Note 3. Protocol for targeted amplification of sgRNAs out of final scATAC-seq libraries.

This supplementary note contains a full protocol for the targeted amplification of sgRNA fragments out of the final 10x scATAC-seq libraries made with Spear-ATAC.

Supplementary Note 1
Protocol for cloning Spear-ATAC plasmids

Background:

- pSP618 was derived from a modified pMJ114 backbone (Addgene # 85995) where the U6-sgRNA cassette was replaced with an alternate sequence from a custom IDT gBlock. This new U6-sgRNA cassette includes a mouse U6 promoter with a 34bp Nextera Read2 adapter in place of the loxP site that is artificially embedded within many mouse U6 promoters used for CRISPR applications, followed by the original constant region (cr1) from the Perturb-seq backbones, followed by a 34bp Nextera Read1 adapter.

- Users can clone sgRNAs into pSP618 in the same way that they might currently clone sgRNAs for pMJ117, pMJ179, or pMJ114 related backbones, or any sgRNA backbone that includes a BstXI site at the 3' end of the U6 promoter and a BlpI site at the 5' end of the sgRNA constant region.

- In this protocol, we outline an example site-directed mutagenesis-based method for inserting individual sgRNA spacer sequences into pSP618. This protocol is only intended to create "end-product plasmids" – you likely do not want to routinely perform whole vector amplification on plasmids that you will modify again in the future because you run the risk of introducing small SNPs/errors that will be propagated to the next generation of plasmids. For example, Q5 high-fidelity polymerase has an approximate error rate of 1bp per 1,000,000bp. However, that means if you are cloning a 10kb plasmid, 1 in every 100 colonies will have an error somewhere within the plasmid – you will not know where unless you sequence the entire plasmid. In general, restriction digest cloning is preferable for long-term plasmids, but site-directed mutagenesis has minimal hands-on time and is a reliable alternative if you also want to use the individual plasmids (separate from the pool).

- For cloning pools of sgRNAs (as opposed to cloning individual sgRNAs as separate plasmids and then pooling the plasmids), we would recommend digesting with BstXI/BlpI and ligating in double-stranded sgRNA spacer inserts.

Reagents:

- 100uM Universal Primer oSP737 (5'-caacaaggtggttctccaagg-3')
- 100uM individual sgRNA-specific Primers (*see below for how to design these primers*)
- pSP618 (Spear-ATAC lentiviral backbone plasmid)
- Q5 Hot Start High Fidelity 2X Master Mix (NEB M0494S/M0494L)
- KLD Enzyme Mix (NEB M0554S)
- NEB 5-alpha Competent E coli (NEB C2987H/C2987P)
- LB plates with 100ug/mL Ampicillin or Carbenicillin
- LB broth with 10ug/mL Ampicillin or Carbenicillin
- QIAprep Spin Miniprep Kit (Qiagen 27104/27106) and/or QIAPrep Spin Midiprep Kit (Qiagen 12943/12945)
- 100uM Sanger sequencing primer oSP652 (5'-gtaatacggttatccacgc-3')

Supplementary Note 1
Protocol for cloning Spear-ATAC plasmids

Designing site-directed mutagenesis primers

sgRNA-specific primers should look like this:

5'-NNNNNNNNNNNNNNNNNNNNgtttaagagctaagctggaaac-3'

Where "NNNNNNNNNNNNNNNNNNNN" is replaced with your sgRNA spacer sequence of interest. Purifying these oligos is not necessary but you might have to screen a couple extra colonies in the end to make sure you get the correct sequence.

Site-directed mutagenesis (SDM)

1. Dilute oSP737 and individual sgRNA-specific primers to 10uM in ddH_2O and dilute pSP618 to 1ng/uL in ddH_2O.

Note: If working with many sgRNA-specific oligos at once, it's easiest to dilute in PCR strips or a 96-well plate (e.g. 5uL 100uM primer to 45uL ddH_2O) and use a multichannel pipette to add 1.25uL of each to the final reactions.

2. Set up the following reaction in PCR strips – scale up according to the number of sgRNAs you are cloning in. Make a master mix and then add 1.25uL of the sgRNA-specific primers to each tube separately. Pipette up and down five times to mix with the pipette set to 20uL.

**reagent**	**volume (x1)**
Q5 Hot Start High Fidelity 2X Master Mix	12.5uL
10uM oSP737	1.25uL
10uM sgRNA-specific Primer	1.25uL
1ng/uL pSP618	2uL
ddH_2O	8uL
total volume:	*25uL*

Note: Here you can also set up negative controls with no sgRNA-specific primer added and/or no pSP618 added.

3. Run the following PCR program:

**temperature**	**time**	**# cycles**
98°C	30"	X1
98°C	10"	X28
62°C	15"	
72°C	4:00	
72°C	2:00	X1
4°C	infinite	X1

Optional STOP: Can leave SDM reactions overnight at 4°C or for several weeks at -20°C if preferred.

4. Run out on gel to ensure the correct product. The correct linearized plasmid product should be ~9kb.

Kinase, ligase, and DpnI incubation

5. Set up the following reaction in PCR strips – scale up according to the number of sgRNAs you are cloning in. Make a master mix and then add 1uL of PCR product from the SDM reaction separately after distributing the master mix (it's easiest to use a multichannel pipette).

**reagent**	**volume (x1)**
KLD Reaction Buffer (2X)	2.5uL
ddH_2O	1uL
KLD Enzyme Mix (10X)	0.5uL
PCR product from SDM reaction above	1uL
total volume:	*5uL*

6. Run the following PCR program:

**temperature**	**time**	**# cycles**
25°C	10:00	X1
37°C	20:00	X1
4°C	infinite	X1

Note: The 37°C step is optional but recommended to further activate DpnI in the KLD Enzyme Mix and prevent parental plasmid contamination in your final product.

Optional STOP: Can leave KLD reactions for several weeks at -20°C if preferred.

Transformation, plasmid preparation, and Sanger sequencing

7. Transform into NEB 5-alpha Competent E coli according to the manufacturer's recommendations, plate out onto LB plates with 100ug/mL ampicillin or carbenicillin, and incubate at 37°C for 14-18 hours.

Note: You can also scale down and add 1uL of KLD product to 10uL of bacteria in PCR strips. Proceed with the rest of the protocol as normal (incubate on ice 30 minutes, 30 second heat shock at 42°C, rest 5 minutes on ice, add 100uL SOC, shake at 37°C for 30 minutes, plate out 50-100uL onto LB plates with 100ug/mL ampicillin or carbenicillin).

8. Pick individual colonies and grow up in 1-5mL of LB broth with 100ug/mL ampicillin or carbenicillin.

Note: For larger experiments, you can grow up colonies in deep 96-well plates with 1mL of LB broth + 100ug/mL ampicillin or carbenicillin per well. Pick between 2-4 colonies for each plasmid to make sure at least one will be the correct sequence.

9. Miniprep according to manufacturer's instructions (Qiagen 27104/27106) or grow up to 20mL for a midiprep.
10. Sanger sequence with oSP652 to check that the new sgRNA spacer sequence is correct.
11. Pool plasmids at an equimolar ratio for your Spear-ATAC experiment and proceed to lentiviral production and transduction (see Supplementary Note 2 for an example protocol).

Note: You might also decide to preferentially weight the non-targeting or safe-targeting control sgRNAs in your pool so that you have extra controls.

Supplementary Note 2
Protocol for making Spear-ATAC lentivirus and sample protocol for transducing cells

Background:

- Since pSP618 was derived from pMJ114 (one of the original Perturb-seq plasmids), it has a polII promoter going in the opposite direction of lentiviral transcription. This design can severely inhibit lentiviral packaging and lead to low lentiviral titers. For performing Spear-ATAC on cell lines, this was not an issue since we were aiming for very low MOIs. However, for cell types that are difficult to transduce, you might consider first cloning the Spear-ATAC sgRNA cassette into a lentiviral backbone without a reverse-facing polII.
- We give a sample protocol for transducing K562;dCas9-KRAB cells; however, transduction will need to be optimized for your cell type of interest.
- Safety note: In addition to transducing your cell type of interest, lentivirus can also transduce your own human cells – please be careful and maintain appropriate biosafety precautions based on your institution and laboratory protocols.

Reagents:

- 293T cells (e.g. ATCC CRL-3216)
- DMEM with 10% FBS (*optional: 1% penicillin-streptomycin and 0.1% amphotericin)*
- 1mg/mL PEI (Polysciences 23966-2)
- Optimem (Thermo 31985062)
- Lentiviral backbone (e.g. pool of pSP618-based Spear-ATAC plasmids)
- VSV-G packaging plasmid (1ug/uL) (e.g. Addgene #8454)
- Delta8.2 packaging plasmid (1ug/uL) (e.g. Addgene #8455)
- 4mg/mL polybrene (Sigma H9268-10G, re-suspended in PBS)

Transfecting 293T cells and making lentivirus

1. Plate 17 million 293T cells in 20mL media on 15cm tissue culture dishes for each virus you are making. Incubate at 37°C with 5% CO_2.
2. ~24 hours later, set up a transfection:

For each 15cm plate:

Transfection reagent mix: 74.25uL 1mg/mL PEI + 1.5mL Optimem
Plasmid mix: 7.4ug lentiviral backbone, 4.95uL delta8.2 (1ug/uL), 1.9uL VSV-G (1ug/uL), 16.5uL Optimem

Mix plasmid mix with reagent mix drop-wise, vortex briefly, incubate at room temperature for 15 minutes, add mix to the 15cm plate of 293Ts drop-wise.

Note: Avoid adding any lentivirus production enhancing agents to the 293Ts during lentiviral production if possible (ex: HDAC inhibitors, etc.) – you run the risk of carrying over these chemicals to your transduced cells and subsequently changing the chromatin landscape.

3. ~24 hours after transfection, change media on the 293Ts. Incubate at 37°C with 5% CO_2.

Note: Be extremely careful as the 293Ts are very loosely attached to the plate. Set your pipette-aid to gravity flow and let the media slowly release onto the plate.

Supplementary Note 2
Protocol for making Spear-ATAC lentivirus and sample protocol for transducing cells

4. ~48 hours after transfection, collect lentivirus-containing media and replace with 20mL fresh media. Incubate at 37°C with 5% CO_2. Keep the 48 hour virus collection at 4°C.
5. ~72 hours after transfection, collect lentivirus-containing media and combine with 48 hour collection. Pass through a 0.45uM filter. Use directly or freeze in aliquots at -80°C until use.

Note: For cells that are difficult to transduce, you can also concentrate the virus at this point. I would recommend using the Lenti-X Concentrator agent from Takara (Takara 631231) – add 1 volume of Lenti-X Concentrator for every three volumes of virus-containing media, chill at 4°C for at least 30 minutes (and up to overnight), and spin down at 1,500g for 45 minutes at 4°C. You should see a white pellet at the bottom of the tube – take off supernatant carefully, and gently re-suspend the white (virus-containing) pellet in 1/10th to 1/100th of the original volume of virus in PBS. Use directly or freeze in aliquots at -80°C until use.

Transducing K562;dCas9-KRAB cells via spinfection

6. Pre-set a centrifuge that can accommodate 6-well plates to 33°C.
7. To each well of a 6-well plate, add 4 million K562;dCas9-KRAB cells, 8ug/mL polybrene, and the appropriate amount of concentrated/unconcentrated virus-containing media. Final volume should be 4mLs per well.

Note: You will need to determine how much virus to add empirically for each batch of virus (i.e. you need to titer your virus). Typically, you will transduce cells with various amounts of virus, check % transduced, and then you can re-do the spinfection with more cells and the appropriate amount of virus per well. For Spear-ATAC, aim for 5-10% of cells transduced to minimize the number of cells transduced with two lentiviruses.

8. Spin at 1,000g for 2 hours at 33°C.
9. Spin off virus-containing media (300g for 5') and re-suspend in 15mL fresh media in T25 flasks.
10. 48 hours post-transduction, check % transduced (BFP+) by FACS.

Supplementary Note 3
Protocol for targeted amplification of sgRNAs out of final scATAC-seq libraries (Spear-ATAC)

Starting material: scATAC library (assuming 150nM—if less, consider scaling # cycles of PCR2 accordingly)

Note: Attempting exponential amplification before streptavidin bead enrichment (or not enriching with streptavidin beads at all) leads to contamination of the scATAC library alongside the sgRNA fragments. The combination of linear amplification beforehand and streptavidin bead enrichment will give you the purest targeted sgRNA library to sequence.

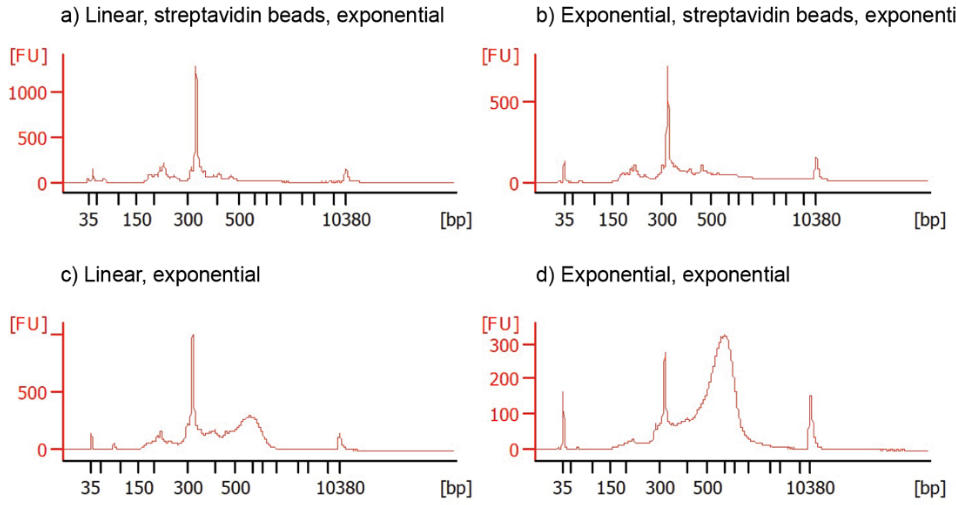

PCR1: Enrich and biotinylate sgRNA fragments in scATAC library (linear amplification)

1. Take 2.5uL of each scATAC library and split it between 8 100uL reactions with oSP2053 (biotinylated, sgRNA-specific primer with TruSeq Read2 adapter attached i.e. linear amplification). Run for 25 cycles with the following parameters:
    - oSP2053: 5'-GTGACTGGAGTTCAGACGTGTGCTCTTCCGATCTaagtatcccttggagaaccaccttg-3'

reagent	X1	X8
**2X NEBNext**	50uL	400uL
**100uM Bio-oSP2053**	0.5uL	4uL
**scATAC library**	0.3125uL	2.5uL
**ddH2O**	49.19uL	393.52uL

**Cycling Conditions**	
98	30"
**Then 25 cycles of:**	
98	10"
63	15"
72	20"
72 2:00; Hold at 4	

2. Pool 8x100uL reactions and PCR purify using Minelute kit. Elute in 40.5uL ddH2O.

Supplementary Note 3
Protocol for targeted amplification of sgRNAs out of final scATAC-seq libraries (Spear-ATAC)

Enrich for biotinylated sgRNA fragments and disenrich scATAC fragments

3. Make B&W buffer. Recipe for 5mL's:
    a. 2mL 5M NaCl
    b. 50uL 1M Tris-HCl pH 7.5
    c. 10uL 0.5M EDTA
    d. 2.94mL ddH2O
4. Vortex Streptavidin MyOne C1 beads 30".
5. Aliquot 25uL beads (per sample) to a PCR strip.
6. Add 200uL B&W buffer and re-suspend by pipetting up and down 5 times with pipette set to 200uL.
7. Magnet 1' on HIGH, discard supernatant.
8. Wash x 2 times more.
9. Re-suspend in 50uL B&W buffer (twice the original volume).
10. Aliquot 40uL of beads to a new tube.
11. Add 40uL of DNA in ddH2O or EB.
12. Incubate for 15' in a thermomixer set at room temperature at 500rpm.
13. Magnet 3' on HIGH.
14. Wash beads 2X with 200uL B&W buffer. Wash 2X with ddH2O.
15. Re-suspend beads in 40uL ddH2O.

PCR2: Add P7/index 1 (exponential amplification)

16. Split the 40uL of PCR1 into 8 100uL reactions with index-specific primer and oSP1594 (P5 specific primer). Run for 15 cycles with the following parameters:
    - oSP1594: 5'- AATGATACGGCGACCACCGAGA-3'
    - Indexed primer: 5'-CAAGCAGAAGACGGCATACGAGAT**NNNNNNNN**GTGACTGGAGTTCAGACGTGTG-3', where **NNNNNNNN** is replaced with the index of choice

reagent	X1	X8
**2X NEBNext**	50uL	400uL
**100uM oSP1594**	0.5uL	4uL
**100uM indexed primer**	0.5uL	4uL
**40uL PCR1 product**	5uL	40uL
**ddH2O**	44uL	352uL

**Cycling Conditions**	
98	30"
**Then 15 cycles of:**	
98	10"
69	15"
72	20"
72 2:00; Hold at 4	

Streptavidin bead disenrichment and clean-up

17. Put back on magnet for 3' on HIGH.
18. Transfer 8x100uL supernatant to fresh tube and PCR purify using Minelute kit. Elute in 20.5uL EB.

KAPA quant and sequence

19. KAPA quant and sequence using the following custom sequencing primer (oMCB1672) for Read1. Alternatively, you can sequence from the Nextera Read1 adapter, but it will be a much longer and expensive read (~150 cycles).

Supplementary Note 3
Protocol for targeted amplification of sgRNAs out of final scATAC-seq libraries (Spear-ATAC)

a. oMCB1672: 5'-GCCACTTTTTCAAGTTGATAACGGACTAGCCTTATTTAAACTTGCTATGCTGTTTCCAGCTTAGCTCTTAAAC-3'
