# Supplementary material for: High-throughput single-cell chromatin accessibility CRISPR screens enable unbiased identification of regulatory networks in cancer
Source: Nat Commun. 2021 May 20;12:2969. doi: 10.1038/s41467-021-23213-w (PMC8137922; doi:10.1038/s41467-021-23213-w)
Supplement: Supplementary file 2 — Reporting Summary [file 41467_2021_23213_MOESM2_ESM.pdf]

nature research

Corresponding author(s): William J. Greenleaf

Last updated by author(s): Feb 8, 2021

Reporting Summary

Nature Research wishes to improve the reproducibility of the work that we publish. This form provides structure for consistency and transparency in reporting. For further information on Nature Research policies, see Authors & Referees and the Editorial Policy Checklist .

Statistics

For all statistical analyses, confirm that the following items are present in the figure legend, table legend, main text, or Methods section.

n/a	Confirmed	
☐	☒	The exact sample size (*n*) for each experimental group/condition, given as a discrete number and unit of measurement
☐	☒	A statement on whether measurements were taken from distinct samples or whether the same sample was measured repeatedly
☐	☒	The statistical test(s) used AND whether they are one- or two-sided<br>*Only common tests should be described solely by name; describe more complex techniques in the Methods section.*
☒	☐	A description of all covariates tested
☐	☒	A description of any assumptions or corrections, such as tests of normality and adjustment for multiple comparisons
☐	☒	A full description of the statistical parameters including central tendency (e.g. means) or other basic estimates (e.g. regression coefficient) AND variation (e.g. standard deviation) or associated estimates of uncertainty (e.g. confidence intervals)
☐	☒	For null hypothesis testing, the test statistic (e.g. *F*, *t*, *r*) with confidence intervals, effect sizes, degrees of freedom and *P* value noted<br>*Give P values as exact values whenever suitable.*
☒	☐	For Bayesian analysis, information on the choice of priors and Markov chain Monte Carlo settings
☒	☐	For hierarchical and complex designs, identification of the appropriate level for tests and full reporting of outcomes
☐	☒	Estimates of effect sizes (e.g. Cohen's *d*, Pearson's *r*), indicating how they were calculated

Our web collection on statistics for biologists contains articles on many of the points above.

Software and code

Policy information about availability of computer code

Data collection

Cell Ranger ATAC 1.2.0 – Barcode Identification, Alignment, Filter, Deduplication

Data analysis

Packages used for customized analysis
fastmatch 1.1-0 - Fast string matching in R.
Biostrings 2.54 - Dictionary string matching in R (PDict)
ShortRead-1.44.3 - For reading fastqs to align sgRNA barcodes.

Packages used for ArchR-related scATAC-seq analysis
ArchR - 0.9.5 - Software for analysis of scATAC-seq data.
macs2 2.1.1.20160309 – Peak Calling
R version 3.6.1 – R environment for all custom code
rhdf5 - 2.30.1 - Software for HDF5 formatted analysis.
Irlba 2.3.3 – Running PCA/SVD on large matrices.
Rcpp 1.0.4 – Used for writing helpful C++ code to speed up operations.
Rtsne 0.15 – Used for t-SNE embeddings.
matrixStats 0.56.0 – Used for mathematical operations on large matrices.
chromVAR_1.8.0 – Calculating TF deviation scores which can be associated with TF activity.
Summarized Experiment 1.16.1 – R Data Class Environment used throughout analyses.
Motifmatchr 1.8.0 – Matching TF Motifs within peak regions
Seurat_3.1.2 – SNN Graph Clustering Implementation
GenomicFeatures 1.32.2 – Genomic Ranges Operations used for overlap analyses
GenomicRanges 1.38.0 - Genomic Ranges Operations used for overlap analyses
Matrix 1.2-14 – Sparse Matrix math implementations.

BSgenome 1.54.0 – Toolkit used for getting Genomic DNA sequences for motif matching and footprinting.
Rsamtools 2.2.3 – For manipulating BAM files within R.
uwot-0.1.5 - For creating UMAPs in R.

Github link- https://github.com/GreenleafLab/SpearATAC_MS_2021

For manuscripts utilizing custom algorithms or software that are central to the research but not yet described in published literature, software must be made available to editors/reviewers. We strongly encourage code deposition in a community repository (e.g. GitHub). See the Nature Research guidelines for submitting code & software for further information.

Data

Policy information about availability of data

All manuscripts must include a data availability statement. This statement should provide the following information, where applicable:
- Accession codes, unique identifiers, or web links for publicly available datasets
- A list of figures that have associated raw data
- A description of any restrictions on data availability

Data availability: We have made available all matrices (peak matrix and chromVAR) available through AWS (See Supplementary Table 6). We also made the 10x cell ranger atac output files and all scATAC-seq matrices used in this study available through AWS. All sequencing data have been deposited in the Gene Expression Omnibus (GEO) at GSE168851 [https://www.ncbi.nlm.nih.gov/geo/query/acc.cgi?acc=GSE168851].

Field-specific reporting

Please select the one below that is the best fit for your research. If you are not sure, read the appropriate sections before making your selection.

[x] Life sciences [ ] Behavioural & social sciences [ ] Ecological, evolutionary & environmental sciences

For a reference copy of the document with all sections, see nature.com/documents/nr-reporting-summary-flat.pdf

Life sciences study design

All studies must disclose on these points even when the disclosure is negative.

Sample size	Sample size was set to make sure results were consistently reproducible. We made sure we captured at least 4000-5000 high-quality cells for each experiment.
Data exclusions	No data were excluded from the manuscript.
Replication	2-3 sgRNAs were used for each target studied. When possible, we included multiple replicates of scATAC-seq data sets to ensure fidelity in the analysis (6 replicates in the Large K562 screen, 4 replicates each for the MCF7 and GM12878 Large screens, 1 replicate for each time-point for the time-course, and 1 replicate for the pilot study).
Randomization	For all experiments, cells/samples were randomly grouped to avoid bias.
Blinding	For in vitro (non-sequencing) experiments, no blinding was performed because experiments were duplicated and triplicated to avoid bias. For sequencing experiments, no blinding was performed because we used multiple sgRNAs for each target and the results had to match between independent sgRNAs to avoid bias.

Reporting for specific materials, systems and methods

We require information from authors about some types of materials, experimental systems and methods used in many studies. Here, indicate whether each material, system or method listed is relevant to your study. If you are not sure if a list item applies to your research, read the appropriate section before selecting a response.

Materials & experimental systems

n/a	Involved in the study	
[x]	[ ]	Antibodies
[ ]	[x]	Eukaryotic cell lines
[x]	[ ]	Palaeontology
[x]	[ ]	Animals and other organisms
[x]	[ ]	Human research participants
[x]	[ ]	Clinical data

Methods

n/a	Involved in the study	
[x]	[ ]	ChIP-seq
[x]	[ ]	Flow cytometry
[x]	[ ]	MRI-based neuroimaging

Eukaryotic cell lines

Policy information about cell lines

Cell line source(s)	Human cell lines (K562, GM12878, and MCF7) were a gift from Michael Bassik and Howard Chang's laboratories, who previously purchased them from ATCC. 293T cells were purchased from ATCC.
Authentication	Cell lines were not directly authentificated but have the morphology/genomic profiles that would be expected given their cell line of origin.
Mycoplasma contamination	All cell lines tested negative for mycoplasma contamination prior to use in experiments.
Commonly misidentified lines (See ICLAC register)	None of the cell lines used in this study are listed in this database.
