# Supplementary material for: High-throughput single-cell chromatin accessibility CRISPR screens enable unbiased identification of regulatory networks in cancer
Source: Nat Commun. 2021 May 20;12:2969. doi: 10.1038/s41467-021-23213-w (PMC8137922; doi:10.1038/s41467-021-23213-w)
Supplement: Supplementary file 3 — Description of Additional Supplementary Files [file 41467_2021_23213_MOESM3_ESM.pdf]

Title: Supplementary Data 1.
Description: Spear-ATAC experiment statistics. This table contains information about each Spear-ATAC dataset generated in this study including QC statistics and number of sgRNA to cell associations captured.

Title: Supplementary Data 2.
Description: Motif accessibility results from K562 pilot Spear-ATAC screen. This table contains information for the sgRNA:TF motif accessibility changes between targeting and non-targeting cells for the K562 pilot screen. Significance is determined based on Chi-Squared adjusted (one-sided) p-values.

Title: Supplementary Data 3.
Description: Motif accessibility results from K562 time-course SpearATAC screen. This table contains information for the sgRNA:TF motif accessibility changes between targeting and non-targeting cells for the K562 time-course Spear-ATAC screen. Significance is determined based on Chi-Squared adjusted (one-sided) p-values.

Title: Supplementary Data 4.
Description: Motif accessibility results from K562, GM12878, and MCF7 transcription factor Spear-ATAC screens. This table contains information for the sgRNA:TF motif accessibility changes between targeting and non-targeting cells for the K562, GM12878, and MCF7 transcription factor Spear-ATAC screens. Significance is determined based on Chi-Squared adjusted (onesided) p-values.

Title: Supplementary Data 5.
Description: List of Vierstra motif clustering annotations used for all motif analyses. This table contains the motif clustering annotations used for motif de-duplication and analyses.

Title: Supplementary Data 6.
Description: Spear-ATAC data URLs. This data contains information for how to access the Spear-ATAC data for each experiment in this study.

Title: Supplementary Data 7.
Description: List of sgRNA spacer sequences used in this study. This table contains information about the sgRNA spacer sequences used in this study and their targets.

Title: Supplementary Data 8.
Description: List of primer sequenced used in this study. This table contains information about key primer sequences used in this study.
